# Heterologous combinations of VSV-GP and native-like trimers elicit autologous Tier 2 HIV antibodies in rabbits

**DOI:** 10.1038/s41541-025-01334-3

**Published:** 2025-12-15

**Authors:** Frederik Radvan, Alexandra Hauser, Li-Yun Lin, Sarah Wilmschen-Tober, Marion Schaber, Marta Bermejo-Jambrina, Tariq Oluwakunmi Agbabiaka, David Peterhoff, Lydia Riepler, Nadja Mendrzyk, Anja Beierfuß, Cornelia Speth, Christiane Moog, Dorothee von Laer, Ralf Wagner, Janine Kimpel

**Affiliations:** 1 Department of Hygiene, Microbiology and Virology, Institute of Virology, Medical University of Innsbruck, Innsbruck, Austria; 2 Institute of Medical Microbiology and Hygiene, University of Regensburg, Regensburg, Germany; 3 Inserm U1109, Institute of Hematology and Immunology, University of Strasbourg, Strasbourg, France; 4 Vaccine Research Institute (VRI), Paris, France; 5 Institute of Clinical Microbiology and Hygiene, University Hospital Regensburg, Regensburg, Germany; 6 Central Laboratory Animal Facility, Medical University of Innsbruck, Innsbruck, Austria; 7 Department of Hygiene, Microbiology and Virology, Institute of Hygiene and Medical Microbiology, Medical University of Innsbruck, Innsbruck, Austria

**Keywords:** Vaccines, Viral vectors, Vaccines

## Abstract

Developing an effective human immunodeficiency virus (HIV) vaccine remains challenging due to difficulties to induce antibodies that neutralize the wide range of HIV-variants. Native-like HIV envelope (Env) trimers in a closed conformation represent promising immunogens. We evaluated the immunogenicity of the chimeric vesicular stomatitis virus-based vector VSV-GP encoding clade C membrane-tethered native-like trimers in heterologous prime/boost combinations with autologous protein. Infected cells displayed high levels of native-like trimers on the surface in a favorable conformation and native-like trimers were also efficiently incorporated into VSV-GP particles. Heterologous vector/protein immunizations outperformed homologous vector regimens, regardless of administration order. In rabbits, these regimens elicited Tier 1 and autologous Tier 2 neutralizing antibodies. Tier 2 neutralization was restricted to pseudoviruses matching the engineered Env-immunogen, with no cross-neutralization of parental Env-variants. Our findings support the use of VSV-GP as a potent platform for displaying native-like Env-trimers and highlight its potential in prime-boost strategies for HIV vaccine development.

## Introduction

Human immunodeficiency virus (HIV) still accounted for about 630,000 deaths in 2023, and more than 1.3 million new infections were registered^1^. According to UNIADS in 2023, 86% of all people living with HIV were aware of their infection, and the majority of those with a known HIV infection received anti-retroviral treatment. However, there is still no cure for HIV infection, and people living with HIV will need life-long treatment with anti-retroviral drugs to prevent viral rebound, AIDS-like disease, and potential transmission of the virus. Despite the increased coverage of anti-retroviral treatment in people living with HIV and recent progress in HIV pre-exposure prophylaxis in those at high risk for infections, the development of an effective preventive vaccine would still be a crucial step in controlling the HIV epidemic^2^. Unfortunately, the development of such vaccines turns out to be a major challenge for scientists globally. Critical issues faced by vaccines are the high mutation rates of HIV, the induction of strain-specific antibodies, and the difficulty to induce durable antibodies with broad neutralization activity^3^. During natural HIV-1 infection, broadly neutralizing antibodies (bnAbs) appear in some individuals, but only after several years^4,5^. In contrast, vaccination regimens shall elicit antibody responses within a short time span and minimize the number of required injections. One option to achieve this is to combine viral vectors and protein immunogens to induce different pathways of the immune system and to circumvent anti-vector immunity, which is observed upon repeated administration of a viral vector^6,7^.

VSV-GP is a chimeric virus based on the vesicular stomatitis virus (VSV), where the glycoprotein G of VSV has been replaced by the glycoprotein GP of the lymphocytic choriomeningitis virus (LCMV)^8,9^. VSV-GP has lost the inherent neurotoxicity of VSV and is therefore an interesting vaccine vector candidate^9^. Another advantage of VSV-GP as a vaccine vector for HIV is that VSV is an enveloped virus that can incorporate HIV envelope (Env) proteins into the virus particle. We showed in a previous study that a chimeric protein consisting of the extracellular part of HIV Env fused to the transmembrane domain and cytoplasmic tail of VSV G was well incorporated into VSV-GP particles and that immunization with a VSV-GP variant encoding membrane-tethered Env induced higher anti-Env antibody titers compared to a VSV-GP vector encoding soluble Env^10^. For that previous study, we used Env of the transmitted/founder virus 1086.C, but the vector only induced Tier 1A neutralizing antibodies in rabbits.

An alternative approach for HIV vaccines is native-like trimer Env proteins, which fold similarly to native Env proteins on the virus during the early docking process to target cells^11^. They allow superior presentation of epitopes for neutralizing antibodies and are stabilized in a pre-fusion state to prevent the dissociation of gp120 and gp41, limiting the induction of strain-specific antibodies^11^. Originally, soluble native-like trimers were stabilized by the SOSIP configuration, which introduces an additional disulfide bridge between gp120 and gp41 together with an I559P mutation to stabilize Env in its pre-fusion conformation^12^. However, HIV Env with SOSIP configuration requires complete furin cleavage for correct folding. For vector-encoded native-like trimers in SOSIP configuration, high basal furin expression in infected cells is crucial for proper processing and trimer formation, but cannot be guaranteed in vivo^13^. Therefore, for vector-encoded Env antigens, an alternative, furin-independent configuration may be preferable. In the native flexibly linked (NFL) configuration, a 10 amino acid flexible glycine-serine linker replaces the furin cleavage site and thereby links gp120 and gp41 permanently^14^. It has been shown that soluble Env antigen in NFL configuration builds well-folded native-like trimers.

Within the European Vaccine Alliance, several clade C-derived soluble native-like gp140 trimers have been developed. Among them is a consensus clade C trimer, which shows improved antigenicity and immunogenicity following stepwise conformational stabilization^15^ and multimerization on nanoparticle carriers^16^. Additionally, two soluble native-like trimers, sC22v4 KIKO* gp140 and sC23v4 KIKO* gp140, have been designed^17,18^. These trimers are stabilized in a pre-fusion SOSIP configuration, with one glycan knocked-in (KI) to restore binding to 2G12 and two glycans knocked-out (KO) to improve accessibility of the CD4 binding side (CD4bs), essentially as described^15^ (Hauser et al. manuscript in preparation).

In this study, we generated VSV-GP vectors expressing membrane-anchored sC22v4 KIKO* and sC23v4 KIKO* variants and characterized folding of Env on infected cells, incorporation of Env into the viral vector and replication of vectors in vitro. Further, we investigated the immunogenicity of the vectors alone or in heterologous prime boost combinations with the corresponding pre-fusion stabilized Env trimer protein immunogens in mice and rabbits. In mice, we found that heterologous prime-boost combinations of VSV-GP-Env and soluble native-like trimers were superior to homologous vector immunizations. In rabbits, heterologous vaccination could evoke heterologous Tier 1 and autologous Tier 2 neutralizing antibodies. Thus, VSV-GP-Env is a promising platform for heterologous prime-boost HIV-1 vaccination regimens.

## Results

### In vitro characterization of VSV-GP vectors expressing HIV-1 Env native-like trimer constructs

We have previously used the viral vector VSV-GP encoding the transmitted/founder Env 1086.C as vaccine antigen, however, this vector induced only Tier 1A antibodies in rabbits^10^. Based on these findings, the current study focuses on next-generation Env native-like trimer antigens.

In a previous study, we showed that Env/VSV-G chimeric proteins were much better incorporated into VSV-GP particles than full-length gp160, and that VSV-GP vectors encoding particle-incorporated HIV Env induced higher titers of Env-specific antibodies than a variant encoding the poorly particle-incorporated full-length gp160 or a soluble gp140 Env^10^. Therefore, in the current study, we also aimed to incorporate the native-like trimer Env antigens sC22v4 KIKO* and sC23v4 KIKO* into the vector envelope. For this reason, we fused HIV gp140 (after position 678) to the transmembrane domain and cytoplasmic tail of the VSV glycoprotein G (position 462–511), resulting in gp140:G fusion antigens as previously described by Bresk et al.^10^. Additionally, a second variant of the fusion protein was generated, where position 462-467 in VSV G were replaced by isoleucine, resulting in gp140:GΔ6 fusion protein. The rational of this modification was to maintain binding to the 10E8 epitope in the membrane-proximal external region (MPER)^18^. Supplementary Table 1 shows the configuration of antigen cassettes used in this study.

**Fig. 1:**
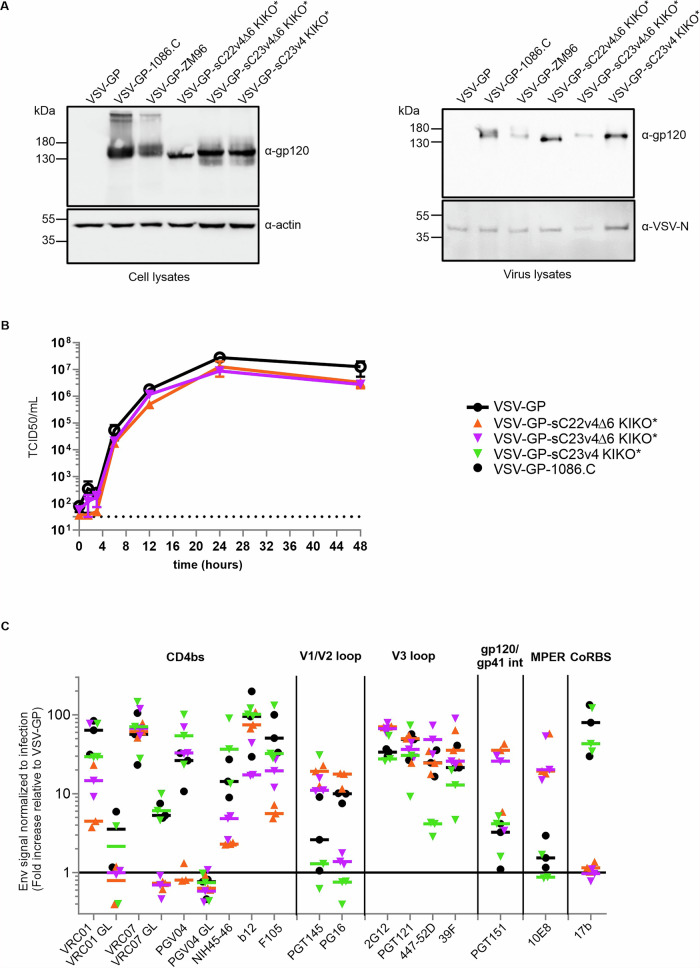
Membrane-anchored native-like trimers are expressed from the viral vector VSV-GP in a favorable conformation and are incorporated into vector particles. **A** HEK293F cells were infected with VSV-GP-Env variants at a multiplicity of infection (MOI) of 0.1, and cell lysates were prepared. Virus samples were generated from sterile-filtrated supernatants of infected 293F cells, followed by gradient centrifugation through sucrose. Presence of proteins was confirmed via Western blotting using 16H3 (anti-gp120), anti-VSV-N and anti-actin antibodies. **B** Single-step growth kinetics for VSV-GP-Env variants or parental VSV-GP without vaccine antigen as control on BHK-21 cells. Viral titers were determined using a 50% tissue culture infectious dose (TCID_50_) assay. Shown are the titrations of duplicates for each virus and time point (mean ± SD). Dotted line indicates limit of detection (3.16 × 10^1^ TCID_50_/ml). **C** 293T cells were infected with VSV-GP-Env variants at an MOI of 0.1, and 24 h post-infection, cells were stained with Env-specific antibodies or the LCMV GP-specific Wen4 as an infection control. Secondary Cy5-coupled anti-human- or APC-coupled-anti-mouse IgG antibodies were used for detection. Binding of human neutralizing antibodies to VSV-GP-Env-infected 293T cells was quantitated by flow cytometry. Env-specific signals were normalized to infection (Wen4) and subsequently expressed as fold-increase relative to VSV-GP. Epitope regions on Env include the CD4 binding site (CD4bs), the V1/V2 loop, the V3 loop, the gp120/gp41 interface, the membrane-proximal external region (MPER) and the CD4-induced co-receptor binding site (CD4i CoRBS). Shown are the results of three independent experiments as individual symbols and the medians as bold lines.

First, we analyzed expression of sC22v4 KIKO* and sC23v4 KIKO* gp140:G fusion constructs with and without the Δ6 modification in VSV-GP-Env-infected cells and incorporation of the chimeric Env antigens into the viral vector particles. The Δ6 modification enhances exposure of the MPER region by replacing amino acids 462–467 in VSV-G with isoleucine, thereby improving accessibility for MPER-targeting broadly neutralizing antibodies. We found that all Env antigens tested were expressed at high levels in infected cells and were well incorporated into the viral particles, similar to the previously described VSV-GP vector encoding 1086.C gp140:G (Fig. 1A and Supplementary Fig. 2).

The introduction of the vaccine antigen cassette into VSV-GP may attenuate the viral vector. To determine whether native-like trimer inserts affect the replication of VSV-GP-vectors, we analyzed the replication kinetics of two VSV-GP-Env variants. However, both VSV-GP-sC22v4∆6 KIKO* and VSV-GP-sC23v4∆6 KIKO* exhibited comparable replication kinetics as the previously described VSV-GP-1086.C^10^ and the empty vector VSV-GP (Fig. 1B).

Soluble sC22v4 KIKO* and sC23v4 KIKO* protein antigens were originally optimized in a SOSIP configuration^15^(Hauser et al. manuscript in preparation). Env antigens with a SOSIP configuration require cleavage at the furin cleavage site for optimal folding, which can be easily guaranteed by furin overexpression in the production cell line when expressing protein antigens in vitro. However, when the vaccine antigen is expressed in vivo upon immunization with the viral vector VSV-GP, it remains unclear whether all infected cells express sufficient amounts of furin to achieve complete cleavage and stabilization of the Env trimer in its pre-fusion conformation. Therefore, a furin cleavage-independent configuration of Env, such as the NFL configuration, might be beneficial for Env expressed from a viral vector. To capture major structural differences resulting from our strategy of linking gp120 and gp41-derived ectodomain via a native flexible linker, we generated VSV-GP variants expressing sC23v4 gp140:GΔ6 KIKO* as NFL and as SOSIP variants and analyzed Env folding on the cell surface of vector-infected cells by flow cytometry (Supplementary Fig. 1A). Both Env variants showed a similar pattern of recognition when using a selection of mostly structure-dependent broadly neutralizing antibodies, suggesting an overall similar structure of the membrane-tethered pre-fusion stabilized Env trimers following infection with recombinant VSV-GP. We therefore continued to use the furin-independent NFL configuration for the delivery of native-like trimers by VSV-GP.

Next, we analyzed the display and overall conformation of the native-like trimers sC22v4 KIKO* and sC23v4 KIKO* on the surface of infected cells in more detail. For VSV-GP-sC23v4 KIKO*, we generated two different Env:G chimeric constructs, gp140:G and gp140:GΔ6, as described above, to analyze if the Δ6 modification indeed enhanced accessibility of the 10E8 epitope as predicted. As control, we used the previously published VSV-GP vector expressing the open trimer 1086.C gp140:G^10^. Infected cells were stained using HIV Env-specific antibodies and analyzed via flow cytometry. Staining for the Env-specific antibodies was normalized to infection using a vector-specific anti-LCMV GP antibody (Fig. 1C). As expected, the 1086.C gp140:G trimer adopted a more open conformation and was well recognized by 17b, which targets a CD4-induced epitope on gp120, while sC22v4Δ6 KIKO* and sC23v4Δ6 KIKO* closed trimers did not expose the 17b epitope. Notably, the Δ6 modification enhanced the binding of 10E8 (MPER) and therefore seems to be a favorable modification for the expression of Env:G chimeric antigens by the vector VSV-GP. The binding profiles of VSV-GP-sC23v4 KIKO* and VSV-GP-sC23v4∆6 KIKO* infected cells were similar regarding most other antibodies. sC23v4 KIKO* was recognized better by the germline antibodies VRC01GL, VRC07GL and PGV04GL (CD4bs), as well as b12 (CD4bs) and 17b (CoRBS). In contrast, superior binding of sC23v4∆6 KIKO* was observed for the antibodies PGT145 (V1/V2 loop), 447–52D (V3 loop), PGT151 (gp120/gp41 interface) and 10E8. Abolished 17b and reduced F105 binding indicate a favorable conformation of membrane-anchored sC23v4Δ6 KIKO* compared to membrane-anchored sC23v4 KIKO*. The antigens sC22v4∆6 KIKO* and sC23v4∆6 KIKO* displayed comparable binding profiles, but differed for PGV04 (CD4bs), where sC23v4∆6 KIKO* was superior, and PG16 (V1/V2), where sC22v4∆6 KIKO* was recognized better. Based on our in vitro characterization, we selected the gp140:GΔ6 constructs with an NFL configuration for in vivo experiments.

### Immunogenicity of VSV-GP-Env with membrane-tethered native-like trimers in mice

**Fig. 2:**
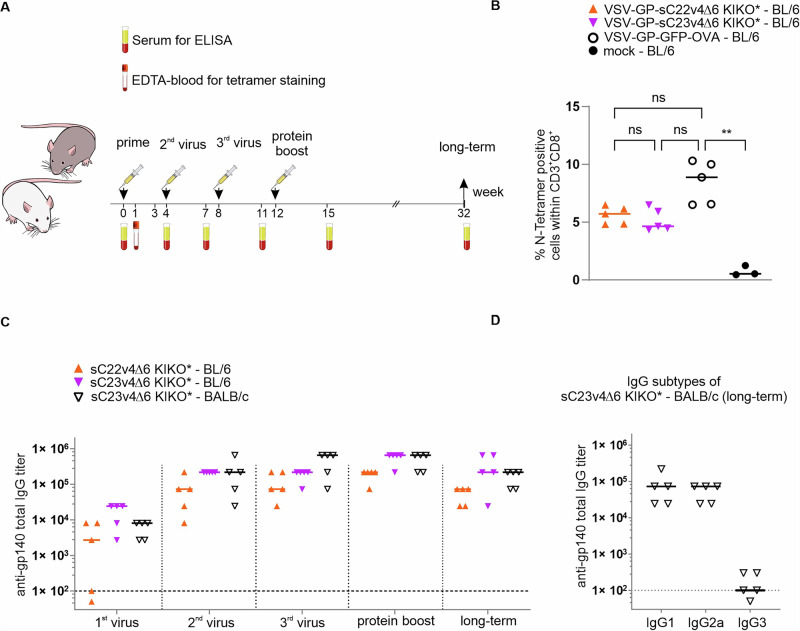
VSV-GP-Env variants carrying native-like trimers are immunogenic in mice. **A** Female C57BL/6JRj or BALB/cRj mice were immunized three times with 1 × 10^7^ TCID_50_ of VSV-GP-Env in week 0, 4, and 8, and were boosted in week 12 with 20 µg of the respective protein plus MPLA. EDTA blood was taken 1 week after the first immunization for tetramer staining, and serum was collected 3 weeks after each and 20 weeks after the last immunization for ELISA. **B** EDTA-blood of C57BL/6JRj mice was collected one week after the first immunization and stained with VSV N-tetramer and antibodies against CD3 and CD8. Percentages of tetramer-positive cells within CD3^+^CD8^+^ T cells were determined via flow cytometry. Individual animals are depicted as symbols, and the median as bold lines. Statistical differences were determined using the nonparametric Kruskal–Wallis test. ns nonsignificant; ** = *p* < 0.01. **C** Endpoint titers of autologous gp140-binding IgG antibodies determined using ELISA. **D** Endpoint titers of autologous gp140-binding IgG-subtype antibodies (IgG1, IgG2a, IgG3) in sera of BALB/c mice at the long-term time point. For **C**, **D** values for individual mice (*n* = 5) and the median are shown. Dotted lines indicate limit of detection (1:100). Values on the dotted line, i.e., titers of 1:100, are considered positive, while values below 100 are considered negative and were set to 50.

As a first step, we assessed the immunogenicity of VSV-GP vectors encoding chimeric sC22v4∆6 KIKO* or sC23v4∆6 KIKO* trimers in mice. Female C57BL/6JRj and BALB/cRj mice received three intramuscular immunizations with 10^7^ TCID_50_ of VSV-GP-sC22v4∆6 KIKO* or VSV-GP-sC23v4∆6 KIKO* with a 4-week interval between doses, followed by one protein boost with 20 µg of matched native-like trimer gp140-SOSIP soluble protein (sC22v4 KIKO* or sC23v4 KIKO*) adjuvanted with 50 µg TLR4 agonist monophospholipid A (MPLA) 4 weeks after the last virus immunization (Fig. 2A).

To compare the general immunogenicity of the different VSV-GP-derived vectors, we first analyzed CD8^+^ T cell responses against the immunodominant epitope RGYVYQGL in the nucleoprotein N in the vector backbone in C57BL/6JRj mice (Fig. 2B). Responses elicited by VSV-GP-sC22v4∆6 KIKO* or VSV-GP-sC23v4∆6 KIKO* were comparable to those animals immunized with a VSV-GP control vector encoding ovalbumin, indicating that the vectors exhibit similar overall immunogenic potential. We next assessed serum binding antibodies against autologous gp140 proteins via ELISA. While in control vector immunized animals, no binding antibodies against gp140 could be detected, all VSV-GP-Env immunized animals developed gp140-binding antibodies latest after the second vector immunization (Fig. 2C). Binding antibodies against autologous gp140 were similar in all three VSV-GP-Env immunized groups, with a tendency of lower titers for VSV-GP-sC22v4∆6 KIKO* immunized mice. All animals receiving VSV-GP-sC23v4∆6 KIKO* showed detectable autologous binding antibodies 3 weeks after the first immunization (1^st^ virus), whilst two animals receiving VSV-GP-sC22v4∆6 KIKO* had antibody titers below the detection limit. The first boost (2nd virus) with VSV-GP-Env increased binding antibody titers in all treatment groups, after which titers plateaued. Long-term samples were collected 20 weeks after the protein boost in week 12. Titers slightly decreased for all groups compared to week 3 after the protein boost, however, high titers were detected in all groups (Fig. 2C). Furthermore, we evaluated long-term samples of VSV-GP-sC23v4∆6 KIKO*/sC23v4 KIKO* protein-immunized BALB/c mice for gp140-binding IgG antibody subclasses IgG1, IgG2a and IgG3. We found that animals mainly produced IgG1 and IgG2a antibodies but only low levels of IgG3 antibodies (Fig. 2D).

### Immunogenicity of different VSV-GP-Env/protein prime-boost regimens in mice

**Fig. 3:**
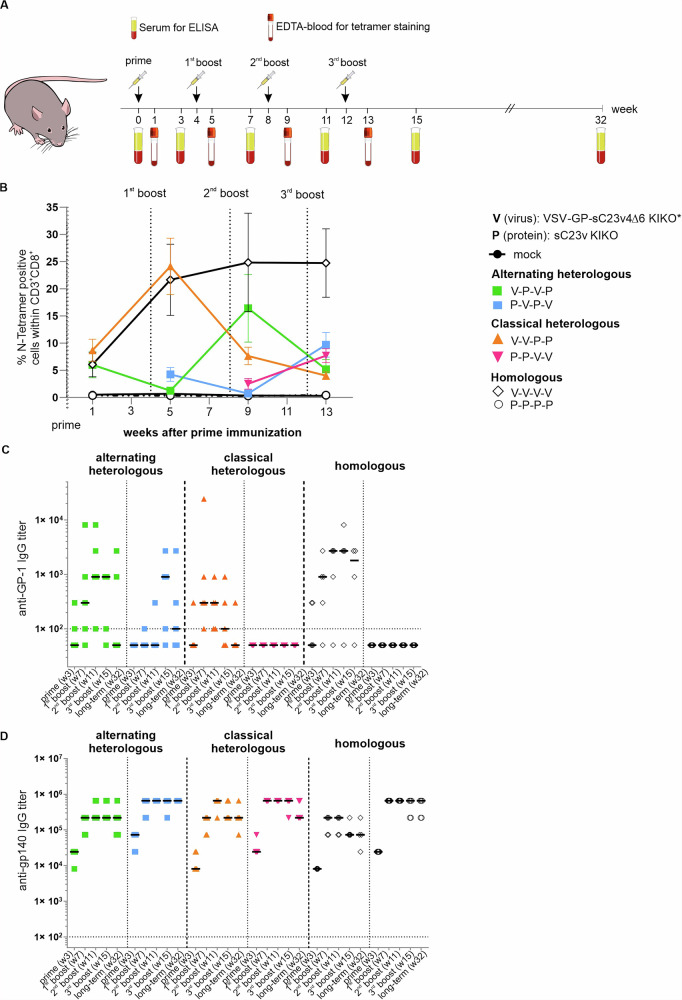
Classical or alternating heterologous regimens of VSV-GP-Env and soluble Env protein induce similar antibody titers. **A** Female C57BL/6JRj mice received four intramuscular injections in weeks 0, 4, 8, and 12 of VSV-GP-sC23v4Δ6 KIKO* (dose 1 × 10^7^ TCID_50_) or 20 µg soluble sC23v4 KIKO* protein plus MPLA in different immunization patterns. One week after each immunization, EDTA-blood was collected for T cell analyses, and 3 weeks after each and 20 weeks after the last immunization, serum was obtained for ELISA. (*n* = 5 per treatment group). **B** Vector-specific T cell responses against the C57BL/6JRj immunodominant RGYVYQGL epitope were determined using tetramer staining and flow cytometry. Shown are N tetramer-positive cells within CD3^+^CD8^+^ T cells. Means and standard deviations are plotted. Dotted vertical lines indicate time points of immunizations. **C** Vector-binding antibodies were determined using an ELISA against LCMV GP-1 subunit. Shown are values for individual animals and the median. **D** HIV gp140-binding IgG antibodies against autologous sC23 KIKO* protein were evaluated using a direct ELISA. Endpoint titers were determined, and median values are indicated by bold lines, individual animals are shown as symbols. In **C**, **D**, horizontal dotted lines indicate the detection limit of 100. Values on the dotted line, i.e., titers of 1:100, are considered positive, while values below 100 are considered negative and were set to 50. *N* = 5 animals are shown per group. V vector; P protein.

As anti-vector immunity often limits the efficacy of homologous boosting with the same viral vector, we next explored different VSV-GP/protein prime/boost regimens using VSV-GP-sC23v4∆6 KIKO* and soluble sC23v4 KIKO* gp140 protein. VSV-GP-sC23v4∆6 KIKO* efficiently incorporates Env into the vector membrane. We do not expect any direct neutralization of the vector mediated by Env-specific antibodies, as the Env on the vector is non-infectious and entry is purely mediated by the LCMV glycoprotein. However, pre-existing Env-binding antibodies could potentially bind to VSV-GP-sC23v4∆6 KIKO* particles and eliminate them by complement-mediated lysis, Fc-mediated phagocytosis or related mechanisms, thereby reducing vector immunogenicity. To analyze if this was the case, we pre-immunized C57BL/6JRj mice twice with gp140 protein. Two protein immunizations induced high titers of gp140-binding antibodies, as seen in an earlier study with a similar construct^18^ and in Fig. 3. Pre-immunized mice were subsequently immunized intramuscularly either with an “empty” VSV-GP or VSV-GP-Env with high amounts of Env on the viral particle. As control, naive mice were immunized with VSV-GP-Env. One week post-immunization, mice were analyzed for N-specific CD8^+^ T cell responses via tetramer staining, and we found no significant difference in percentages of anti-vector CD8^+^ T-cells among groups (Supplementary Fig. 1B). These results indicate that VSV-GP vectors with Env incorporated into the virus particle can be used to boost immunity in animals with pre-existing Env-specific antibodies.

We next compared a classical heterologous immunization schedule with two doses of vector followed by two doses of protein or vice versa (V-V-P-P or P-P-V-V), an alternating heterologous schedule (V-P-V-P or P-V-P-V) and homologous combinations (V-V-V-V or P-P-P-P) and hypothesized that heterologous immunization would elicit superior responses compared to four doses of vector, as anti-vector immunity might limit homologous boosting. Peripheral blood was collected one week after each immunization for assessment of anti-vector T cell responses and three weeks after each immunization serum for the analysis of antibody responses (Fig. 3A). CD8^+^ T-cells responses against the VSV-nucleoprotein (N) were induced upon immunization with VSV-GP-sC23v4∆6 KIKO*, but not when protein immunogens were injected (Fig. 3B). As expected, anti-VSV-N T-cells decreased over time but were boosted again after each virus immunization. Increases in anti-vector T-cells were most pronounced when subsequent doses of VSV-GP-sC23v4∆6 KIKO* were administered, reaching up to 25-30% of all CD8^+^ T-cells in the homologous vector immunization group (V-V-V-V).

Moreover, we examined titers of anti-vector antibodies in samples collected 3 weeks after each immunization. Antibodies directed against LCMV GP were quantified using ELISA. Overall, the three animal groups that were primed with VSV-GP-sC23v4∆6 KIKO* developed higher anti-vector antibody titers than those primed with sC23v4 KIKO* protein. As observed for T-cell responses, boosting with VSV-GP-sC23v4∆6 KIKO*, but not sC23v4 KIKO* protein, enhanced anti-vector antibody titers. Interestingly, none of the animals in the P-P-V-V group developed detectable levels of anti-vector antibodies over the monitored time period. At the long-term time point, anti-GP1 antibodies had decreased again in many animals (Fig. 3C).

Further, we evaluated gp140-binding antibody levels in serum samples three weeks after each immunization and additionally 20 weeks after the last immunization. Kinetics of antibody titers were similar among the different vaccination schedules and plateaued after the second immunization (Fig. 3D). In the P-V-P-V group, Env-specific antibodies were boosted by the first vector dose, indicating again that in animals with pre-existing Env-specific antibodies, the VSV-GP vector with Env on the surface was still effective. However, four-dose vector-immunized animals tended to have lower titers of anti-gp140 antibodies than other groups, indicating that vector-only schedules may not be ideal. Antibody titers were stable even 20 weeks after the last immunization in all heterologous immunized groups and were similar in heterologous groups and the homologous protein group. Median antibody levels at the long-term time point did not significantly differ between the heterologous treatment groups and the protein-only group (Fig. 3D). These data suggest that all heterologous immunization patterns combining VSV-GP-Env and soluble Env protein induce strong and durable antibody responses, supporting their potential utility in vaccination approaches. As antibody titers plateaued already after the second immunization for most groups, it is difficult to further differentiate between regimens.

### Immunogenicity of VSV-GP-Env/protein prime-boost regimens in rabbits

**Fig. 4:**
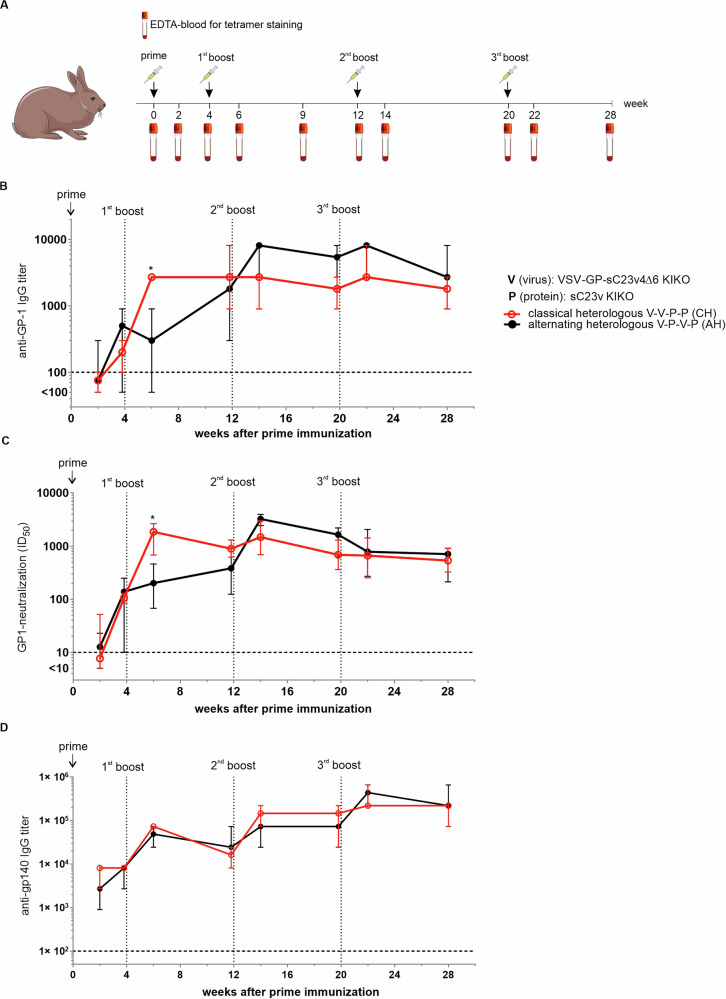
Classical and alternating heterologous combinations of VSV-GP-sC23v4Δ6 KIKO* and soluble sC23v4 KIKO* native-like trimer protein induce high titers of gp140-binding antibodies in rabbits. **A** Female New-Zealand White rabbits received four intramuscular immunizations in weeks 0, 4, 8, and 20 with VSV-GP-sC23v4Δ6 KIKO* (dose 2 × 10^8^ TCID_50_) or sC23v4 KIKO* protein (dose 40 µg protein + 20 µg MPLA) in different immunization patterns (*n* = 4 per treatment group). Two weeks after each immunization, directly prior to each immunization and additionally eight weeks after the last immunization, serum was collected. **B** Vector-binding antibodies were determined using an ELISA against LCMV GP-1 subunit. The horizontal dotted line indicates the detection limit of 100. Samples below the detection limit were set to 50. Shown are median ± 95% confidence interval (CI). **C** Titers of vector neutralizing antibodies (ID_50_) as determined via a VSV-pseudovirus neutralization assay. Shown are the median ± 95% CI of reciprocal ID_50_ titers. The horizontal dotted line indicates the detection limit of 10. **D** HIV gp140-binding IgG antibodies against autologous sC23v4 KIKO* protein were evaluated using a direct ELISA. Endpoint titers were determined, and the median ± 95% CI is shown. Horizontal dotted lines indicate the detection limit of 100. V vector, P protein. Statistical differences between the two groups were determined for each time point using a non-paired, nonparametric t-test. * *p* < 0.05, all other comparisons were nonsignificant.

Since the assessment of HIV neutralization in mice is difficult due to nonspecific inhibition of mouse serum in in vitro neutralization assays, we finally compared two different heterologous vaccination regimens in a rabbit model. We selected two heterologous regimens combining a vector prime with a protein boost for the rabbit study, as a vector prime/protein boost regimen was also used in the partially effective RV144 trial. In particular, we compared a classical heterologous schedule consisting of two vector doses followed by two protein doses (V-V-P-P) and an alternating heterologous schedule with virus and protein alternating with each immunization (V-P-V-P). To allow efficient induction of memory responses, we increased intervals between immunizations and vaccinated rabbits in weeks 0, 4, 12, and 20 (Fig. 4A). New Zealand White rabbits were immunized intramuscularly with 2 × 10^8^ TCID_50_ of VSV-GP-sC23v4Δ6 KIKO*or 40 µg sC23v4 KIKO* gp140 protein plus 20 µg MPLA and serum was collected early (two weeks) and late (1 day prior to the next or 8 weeks after the last immunization) after each immunization to analyze humoral responses against the vector and HIV Env. All immunizations were well tolerated.

Both vaccination schedules elicited similar vector-binding and vector-neutralizing antibodies (Fig. 4B, C). A major difference between the two groups was only observed two weeks after the second immunization, where anti-vector antibody titers were higher in the group that received two vector immunizations compared to the group with one vector and one protein immunization. Finally, we evaluated binding antibody responses against HIV-Env. Autologous binding antibodies against sC23v4 KIKO* were assessed using a direct ELISA. Both vaccination regimens induced comparable binding antibodies against gp140 (Fig. 4D). All animals developed binding antibodies after the prime immunization with VSV-GP-sC23v4∆6 KIKO*. The 1st boost with VSV-GP-sC23v4∆6 KIKO* or sC23v4 KIKO* protein led to a titer increase in both groups. A slight drop in antibody levels was observed 8 weeks after the 1st boost. The 2nd boost enhanced anti-gp140 antibody titers in both groups. Antibody titers remained stable until the last immunization. The final boost (3rd boost) resulted in similar peak antibody titers for both groups. At the long-term time point, median antibody titers of both groups were identical and were comparable to those 2 weeks after the 3rd boost, suggesting decent antibody durability (Fig. 4D). Direct coating of the Env protein onto the plates may result in exposure of epitopes against non-neutralizing antibodies at the back of the trimer or due to loss of the native-like trimer conformation^19^. We therefore re-analyzed sera from the final bleeding of the rabbits using a Ni-NTA capture ELISA. Titers for the capture ELISA were as expected around one log lower, but overall patterns for both assays were similar, and no differences between the two different immunization regimens were seen (Supplementary Fig. 3). This is in line with our previous data^18^.

**Fig. 5:**
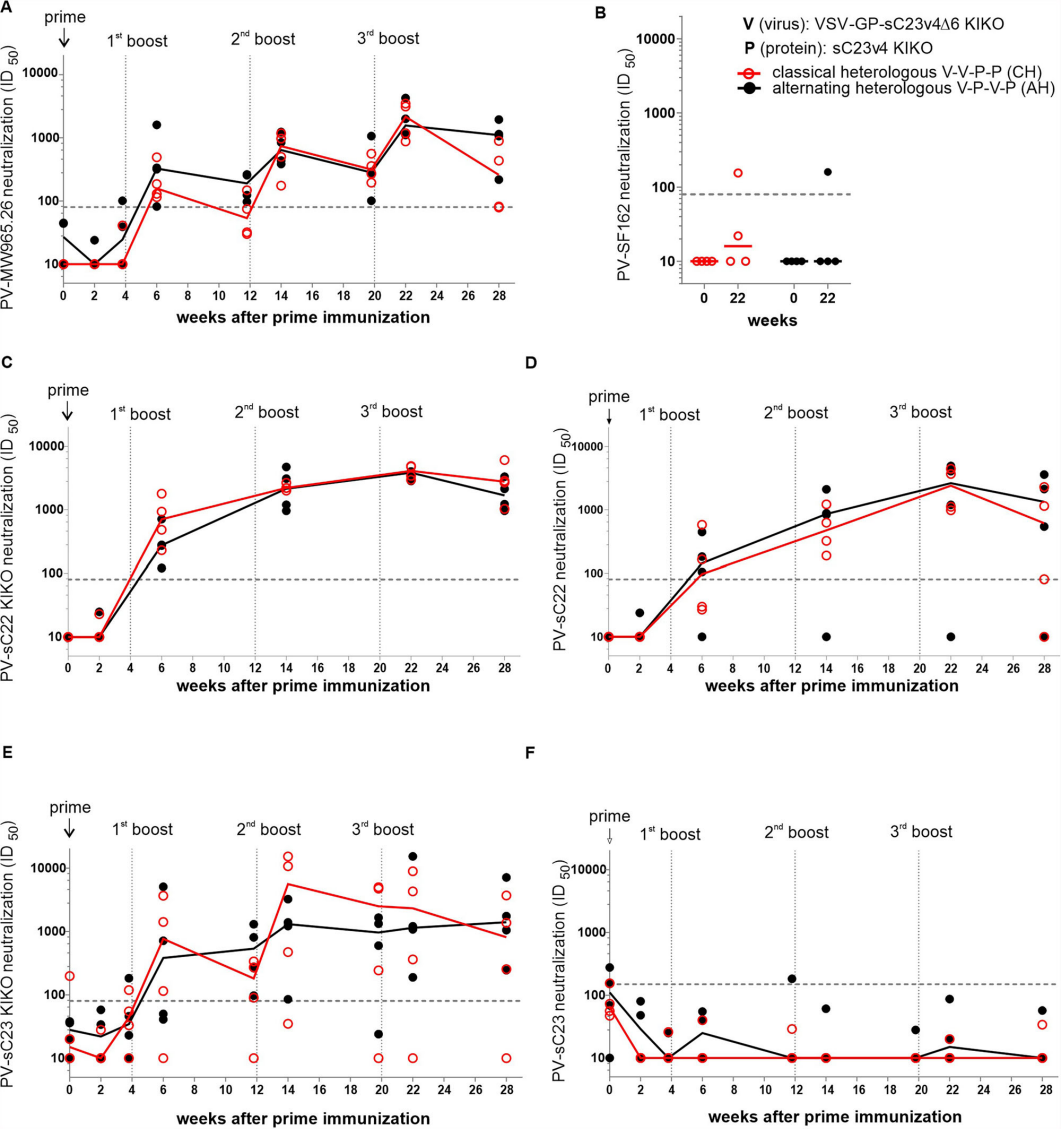
Combinations of VSV-GP-sC23v4Δ6 KIKO* and soluble sC23v4 KIKO* protein induce Tier 1 and autologous Tier 2 neutralizing antibodies in rabbits. Neutralizing antibodies against indicated HIV strains (**A** MW965.26, **B** SF162, **C** sC22 KIKO, **D** sC22, **E** sC23 KIKO, **F** sC23) were determined using a lentivirus pseudovirus assay. Reciprocal ID_50_ titers are plotted, showing individual animals as symbols and the group median as the continuous line (*n* = 4 per group and time point). Dotted horizontal lines indicate the limit of detection (based on pre-immune sera). Dotted vertical lines indicate time points of immunizations. V vector, P protein.

In a last step, we analyzed the functionality of antibody responses. Neutralizing antibodies in rabbit sera against the Tier 1A clade C Env MW965.26, the Tier 1 clade B Env SF162, sC22 KIKO, sC22, and the autologous Env sC23 KIKO and sC23 were assessed using the conventional TZM-bl pseudovirus (PV) neutralization assay^20^. PV-sC22 KIKO, PV-sC22, PV-sC23 KIKO and PV-sC23 were first characterized according to their sensitivity to neutralization using a selected panel of HIV infected human sera and well-known classified Tier PVs as previously performed (Supplementary Fig. 4)^21^. The new PV-sC22 and PV-sC22 KIKO were ranked as Tier 1 easy-to-neutralize viruses, while PV-sC23 and PV-sC23 KIKO were ranked as Tier 2 difficult-to-neutralize viruses (Supplementary Fig. 4). PVs displaying the KIKO variants are more accessible for neutralizing antibodies compared to their non-modified parental counterparts sC22 and sC23, as they have a knock-in of glycans to restore binding to 2G12 and a knock-out of glycans to improve accessibility of CD4bs^15,22^. We found that the kinetics of neutralization profiles were similar between the two immunization regimens for all tested PVs (Fig. 5). All animals developed Tier 1 neutralizing antibodies against PV-MW965.26 as early as 2 weeks after the second immunization. PV-MW965.26 neutralizing antibody titers were slightly boosted with subsequent immunizations and remained positive 8 weeks after the last immunization when the animals were sacrificed. In contrast, no neutralizing antibodies against the clade B Env SF162 were detected. Both KIKO variants, PV-sC22 KIKO and PV-sC23 KIKO, were well neutralized by most of the rabbit sera, with a tendency of better neutralization of PV-sC22 KIKO (heterologous, Tier 1) compared to PV-sC23 KIKO (autologous neutralization, Tier 2). As expected, neutralizing titers against the parental non-modified PV-sC22 and PV-sC23 were lower compared to their KIKO counterparts. Interestingly, the parental PV-sC22 (Tier 1) was still well neutralized, while hardly any neutralizing antibodies against the parental PV-sC23 (Tier 2) were detected.

Taken together, our data indicate that heterologous VSV-GP-Env vector/protein combinations using next-generation native-like trimer antigens induce good and durable antibody responses and might be superior to vector alone immunizations.

## Discussion

Numerous HIV vaccine candidates have been explored in clinical trials, however, none of these have shown efficacy, justifying further market authorization. In previous studies, we described VSV-GP as a promising HIV vaccine vector, but only Tier 1 neutralizing antibodies were induced upon immunization of rabbits^10^. In the current study, we aimed to improve this existing VSV-GP HIV vaccine vector in two ways. The vector was combined with a protein in heterologous prime/boost regimens to reduce anti-vector immunity, which has been shown to limit homologous boosting for various vectors. Indeed, Env-specific antibody titers were higher for VSV-GP/soluble protein heterologous combinations compared to homologous vector immunizations alone. As a second improvement, we incorporated next-generation native-like trimers as the Env antigen into the vector instead of the open trimers used previously. We showed that this combined approach induced Tier 1 and autologous Tier 2 neutralizing antibodies in rabbits.

Many HIV vaccine studies indicate a benefit of heterologous prime/boost regimens using viral vectors and proteins for the induction of high antibody titers^6,23–26^. As protein vaccination alone mainly induces antibody and CD4^+^ T cell responses, the combination of protein-based vaccination strategies with viral vectors, which are known to also induce potent CD8^+^ T cell responses against encoded antigens, may be beneficial for the development of an effective HIV vaccine^27^. Interestingly, also the RV144 trial, the only HIV vaccine clinical trial that so far showed moderate transient efficacy in preventing infections, combined a vector and a protein component in a heterologous schedule^28^. Viral vaccine vectors such as VSV or adenoviral vectors often induce high titers of vector-neutralizing antibodies already after the first immunization, limiting immunogenicity of a second dose with the same vector. One rational for the exchange of VSV’s glycoprotein G by the glycoprotein GP of LCMV in the chimeric VSV-GP vector had been the reduced induction of LCMV-neutralizing antibodies after natural or experimental LCMV infection. In line, a recombinant replication-defective LCMV vector induced only occasionally and transiently vector-neutralizing antibodies after three subsequent immunizations in a clinical trial^29^. VSV-GP induced no vector-neutralizing antibodies in previous experiments in mice^9,30^. In the current study, we observed only moderate vector-neutralizing antibody titers in the rabbits after the first vector dose, indicating that a second dose was still effective. This is supported by an increase of HIV-neutralizing antibodies by the second vector dose for both immunization schedules in our rabbit study. However, other components of the immune system, such as binding antibodies that activate the complement system or anti-vector T cells, might still limit subsequent homologous immunizations with VSV-GP^31,32^. We have previously shown in mouse models that a heterologous combination of VSV-GP with a poxvirus vector or a peptide enhanced vaccine antigen-specific immunity when used as HIV or tumor vaccine, respectively^31,33^. In this study, we compared a classical prime/boost regimen with two doses of vector prime followed by two doses of protein boost with a schedule alternating vaccine platforms with each immunization, i.e., vector-protein-vector-protein. The rationale behind this was to increase the time between the two vector immunizations and to allow boosting of the HIV-specific immune response by a protein immunization prior to the second vector immunization. However, both schedules led to a comparable quantity and quality of Env-specific antibody responses.

As the development of HIV vaccines faces several hurdles due to inherent characteristics of the virus, one crucial factor for success will be the careful selection and optimization of Env antigens. Stabilized native-like trimers mimic HIV-1 Env in a state before binding to a target cell and were shown to induce neutralizing antibodies^11,14,34^. Recently, native-like BG505 trimers have been explored in clinical trials in humans. Autologous Tier 2 neutralizing antibodies were not detected in serum using conventional neutralization assays but could only be isolated from B cells of a vaccinated individual^35^. In that clinical trial, most trimer-binding antibodies targeted the glycan holes at the trimer base^36^. On the other hand, membrane-anchored Env constructs optimized for efficient expression on a viral vector, such as the constructs described by us here and in a previous study^18^, may have the disadvantage of heterogeneity in antigen folding and potentially exposing new artificial epitopes at the Env:G interface. To circumvent this problem, heterologous combinations of membrane-bound Env delivered by a viral vector or nanoparticles with soluble protein or guiding principle with trimers or gp120 core fragments engineered for binding to germline BCR versions of bnAbs might be advantageous. There may be advantages and disadvantages for both combinations, i.e., priming with a vector-delivered membrane-anchored Env followed by a protein boost or vice versa. For the current study, we selected strategies with vector prime and protein boost for the rabbit study, as we believed that boosting with a highly polished native-like trimer may help boost antibodies against epitopes relevant for protection.

Both antigens described here, sC22v4 KIKO* and sC23v4 KIKO*, were originally designed and carefully selected to enable folding of soluble gp140 proteins as well as organized closed native-like trimers^15^(Hauser et al. manuscript in preparation). The high degree of stabilization and V3 shielding of the sC23v4 KIKO trimer has been confirmed in a recent antigenicity screening^17^. Delivery of vaccine antigens via viral vaccine vectors or, as more recently described delivery, via mRNA in lipid nanoparticles allows high expression of vaccine antigens in vivo, however, it also poses an additional hurdle of correct folding of Env antigens, as different types of target cells might be transduced and produce the vaccine antigen in vivo. Soluble protein antigens are expressed in vitro under conditions to allow favorable folding, such as complete furin cleavage for SOSIP variants and are subsequently polished by affinity purification and size exclusion chromatography for selection of native-like, closed-conformation trimers. For Env antigens expressed in vivo, such favorable conformations cannot be guaranteed. Therefore, cleavage-independent conformations that do not depend on a certain amount of furin for quantitative Env processing may be advantageous. Indeed, cleavage-independent NFL and cleavage-dependent SOSIP variants adopted similar conformations when expressed as membrane-anchored sC23v4 KIKO* from the viral vector VSV-GP. Similarly, others explored an alternative cleavage-independent conformation of uncleaved pre-fusion optimized Env trimers for delivery via self-amplifying mRNA or an integrase-defective lentiviral vector^37–39^.

The native-like trimer sC23v4 KIKO* was selected to analyze functional antibody induction in rabbits. Neutralizing antibodies in the rabbit sera were determined via a classical lentiviral PV assay using full-length Env. This assay should therefore be well-suited to detect neutralization relevant for circulating or lab-adapted HIV strains. We found that heterologous vector/protein immunizations (both with sC23v4 KIKO* vaccine antigen) induced autologous Tier 2 neutralizing antibodies against PV-sC23 KIKO, but no neutralizing antibodies against PV-sC23 containing the parental Env. The rational for removing glycans around the CD4bs (N276 and N460/N463) in the KIKO modified Env was to allow better accessibility of the CD4bs for bnAbs such as VRC01 and NIH44-45 and thereby potentially facilitate induction of CD4bs-specific antibodies^40^. Our data indicate that indeed neutralizing antibodies against the engineered CD4bs may have been induced in the rabbits, while these antibodies failed to neutralize PVs bearing the parental sC23 Env, in which the CD4bs remains shielded by its natural glycan complement. However, differences in neutralization of the rabbit sera by PV-sC23 and PV-sC23 KIKO could also be explained by an altered V5 loop between the two Envs or the addition of N295/N448 glycans to restore 2G12 binding. To answer this question, further studies on mapping or isolating and characterizing the induced antibodies should be done.

Our data are in line with a recent clinical trial evaluating a CD40-targeting clade C 96ZM651 immunogen. In that study, sera from immunized participants were also better neutralized by PV-sC22 KIKO with the more accessible CD4bs compared to PV-sC22 with an unmodified CD4bs^41^. Schorcht et al. showed that a clade B-based native-like trimer could induce neutralizing antibodies against the CD4 binding site at least in one of the rabbits^42^. It is interesting to note that neutralizing antibodies against the parental sC22 variant were only slightly lower than against the KIKO-modified sC22 variant with the better accessible CD4bs. This might be explained by epitopes of the induced neutralizing antibodies outside the CD4bs, such as V3 or the gp120/gp41 interface, probably triggered by a slightly more open conformation of the sC22 variants compared to the sC23 variants. PV-sC22 has, in line with these observations, been classified as Tier 1 virus, while PV-sC23 as Tier 2.

One limitation of our study is that only autologous Tier 2 neutralizing antibodies against a glycan-modified vaccine antigen were induced, but not against the parental Env without the glycan hole. In future studies, it should be interesting to combine prime immunizations using KIKO antigens with a boost with the non-modified versions to see if guiding of antibody development is possible. A second limitation is that no direct comparison of our constructs/regimens with a different well-established HIV vaccine platform, such as the BG505 trimers, has been performed. HIV vaccine platforms/antigens that can reliably induce Tier 2 neutralizing antibodies are still limited and factors needed for induction of such bnAbs poorly understood. Future studies directly comparing different vaccine platforms may help to overcome these limitations.

Overall, the VSV-GP vector platform should be a good combination partner for soluble native-like trimers in heterologous immunization schedules as it presents the Env antigen in a potentially favorable conformation on the membrane.

## Methods

### Cell lines

BHK-21 cells (American type culture, Manassas, VA, USA) and BHK-21 cells stably expressing LCMV-GP (in-house generated) were cultivated in Glasgow minimum essential medium (GMEM; Gibco, Carlsbad, CA, USA) supplemented with 10% heat-inactivated fetal calf sera (FCS; Gibco, Life Technologies, Carlsbad, CA, USA), 5% tryptose phosphate broth (Gibco, Carlsbad, CA, USA), 100 units/ml penicillin (Gibco, Carlsbad, CA, USA) and 0.1 mg/ml streptomycin (Gibco, Carlsbad, CA, USA). BHK-21 cells stably expressing LCMV-GP were selected with 10 µg/ml puromycin. 293 T cells (American Type Culture Collection), 293F cells (Gibco, New York, USA) and 293 cells expressing LCMV GP-1 fused to human IgG1 Fc (GP-1-IgG cells, kindly provided by Daniel Pinschewer through European Virus Archive)^43^ were cultured in Dulbecco’s Modified Eagle’s Medium (DMEM; Lonza, Walkerville, MD, USA) supplemented with 10% heat-inactivated FCS, 2 mM L-glutamine, 1× non-essential amino acids (Gibco, Carlsbad, CA, USA), 1 mM sodium pyruvate (Sigma-Aldrich, USA), 100 units/ml penicillin and 0.1 mg/ml streptomycin. 293F suspension cells were cultivated in CD293 medium (FCS-free) (Gibco). Vero cells (ECACC, cat# 08011101) were cultured in DMEM supplemented with 10% heat-inactivated FCS, 2 mM L-glutamine, 100 units/ml penicillin and 0.1 mg/ml streptomycin. The TZM-bl cell line is derived from a HeLa cell clone that was engineered to express CD4, CCR5 and CXCR4 and to contain integrated reporter genes for firefly Luc and *E. coli* β-galactosidase under the control of an HIV-1 long terminal repeat, permitting sensitive and accurate measurements of infection^44,45^.

### Recombinant soluble HIV-1 Env proteins

The sC23v4 KIKO* antigen has been previously described^17,18^. sC22, sC22 KIKO, sC23 and sC23 KIKO have been previously used for generation of PVs^41^. The following briefly describes the rational in the design of these constructs, which is based on principles we previously applied for a clade C consensus native-like trimer^15^. The coding sequences of the two pre-fusion stabilized clade C Env trimers sC22v4 KIKO* and sC23v4 KIKO* used in this study were derived from patient isolates (GenBank accession: KF766537 and AY255826). Clade C sequences were selected due to their high prevalence globally, and the specific sequences based on their probability to form highly stable trimers. Both Env trimers were designed as soluble gp140 proteins with SOSIP configuration (disulfide bridge between A501C and T605C plus I559P mutation) with the approach presented previously^12,15,18^. To further stabilize the trimers in a pre-fusion state we applied design rules initially suggested by Guenaga et al^46^. and modified by Hauser et al.^15^. Additionally, missing N-linked glycosylation sequons (NXS/T) for glycosylation at position N295 and N448 to enable binding of bnAb 2G12 were KI, if not naturally present^47^ and N-linked glycans at positions N276 and N460/N463 around the CD4 binding site were deleted by substitution of asparagine with glutamine (KO)^40^. The resulting pre-fusion stabilized clade C Env trimers are in the following referred to as sC22v4 KIKO* and sC23v4 KIKO*. Prefusion-stabilized Env trimers were produced and purified as described previously^48^.

### Recombinant VSV-GP constructs

VSV-GP, VSV-GP-OVA, VSV-GP-1086.C (VSV-gp140:G-linker), and VSV-GP-ZM96 gp140:G* were described previously^8–10,31^. VSV-GP vectors containing native-like trimer Env antigens as membrane-anchored gp140: G fusion proteins on position 5 in the viral genome were generated as followed. Sequences coding for the different HIV Env variants were amplified by PCR using overlapping sequences to the target vector. VSV-GP-luciferase containing an additional intergenic region and luciferase at position 5 between GP and L^49^, and was digested with NheI and XhoI and HIV Env antigen cassettes were inserted into the vector to replace luciferase using Gibson assembly. VSV-GP-sC23v4 KIKO* expressed a membrane-anchored gp140:G version generated by fusing gp140 after position 678 to the transmembrane domain and cytoplasmic tail of VSV G (position 462–511) as described previously^10^. Alternatively, positions 462–467 of VSV-G were replaced by an isoleucine to enhance accessibility of the 10E8 epitope, resulting in gp140:G∆6 variants and the vectors VSV-GP-sC22v4Δ6 KIKO* and VSV-GP-sC23v4Δ6 KIKO*. The majority of Env antigens in the VSV-GP vector were expressed in NFL configuration^18^. For the sC23v4Δ6 KIKO* antigen, an additional vector with SOSIP was generated to compare folding of Env in infected cells, resulting in the vector VSV-GP-sC23v4Δ6 KIKO* (SOSIP). See Supplementary Table 1 for details regarding VSV-GP-Env vectors used in this study. Rescue of viruses was performed on 293T cells using reverse genetics with a helper virus-free protocol, which was previously established^50^. Rescued viruses were passaged on BHK-21 cells until considerable cytopathic effect was observed, and were subsequently plaque-purified twice on BHK-21. Experimental virus stocks (passage 3 after plaque purification) were produced by infecting BHK-21 cells at an MOI of 0.01. About 24–30 h post infection, supernatants were harvested, filtrated through 0.45 µm filters and concentrated by overnight low-speed centrifugation through a 20% sucrose cushion (Merck, Germany). After removing the supernatant, the virus pellet was re-suspended in PBS and aliquots were frozen at −80 °C. Viral titers were determined with a TCID_50_ assay. The replication-deficient VSV-*∆*G-SEAP virus was generated by replacing VSV G by the secreted alkaline phosphatase (SEAP). The virus was produced on BHK-21 cells stably expressing LCMV GP to generate single-round infectious particles pseudotyped with LCMV GP for neutralization assays.

### TCID_50_ assay

Virus titers for VSV-GP particles were determined via TCID_50_ (50% tissue culture infectious dose) assay. Briefly, 3 × 10^3^ BHK-21 cells per well were seeded in 96-well plates (Sarstedt, Nümbrecht, Germany) and incubated overnight. Semi-logarithmic dilutions (experimental virus stocks) or logarithmic dilutions (replication kinetic) of virus samples were prepared and added to the wells in 8 replicates. Plates were then incubated for 6 days at 37 °C, 5% CO_2_. Finally, plates were visually analyzed for cytopathic effect, and TCID_50_ titers were calculated according to the formula of Spearman and Kaerber.

### Western blot

293F cells were infected with VSV-GP-Env viruses or VSV-GP at an MOI of 0.1. Twenty-four hours post-infection (hpi), cells suspensions were collected in Falcon tubes and centrifuged at 1600 rpm for 5 min. Cell pellets were re-suspended in 200 µl lysis buffer (50 mM HEPES, pH 7.5, 10 mM Na_4_P_2_O_7_ x 10 H_2_O, 150 mM NaCl, 10% Glycerol, 1% Triton X-100, 1 mM Sodium metavanadate, 2 mM EDTA pH 8.0, 2 mM PefablocRSC, 50 mM NaF) on ice for 30 min. Samples were transferred to Eppendorf tubes and centrifuged at 8000 rpm for 20 min at 4 °C. Supernatants were collected and stored at −20 °C. Cell lysates and virus samples (P3 stocks) were diluted in 4X Lämmli buffer (Bio-Rad, California, USA) and denatured at 95 °C for 5 min. Protein bands were separated with SDS-PAGE and transferred to nitrocellulose using a semi-dry blotting system (Transblot SD, Bio-Rad). After blocking the membranes with 5% (w/v) fat-free milk powder in PBST for 2 h, membranes were incubated with respective primary antibodies overnight at 4 °C or for 1 h at room temperature: 16H3 (anti-gp120, 0.2 µg/ml; NIH HIV Reagent Program^51^,), anti-actin (1:5000; Sigma-Aldrich, Missouri, USA) or anti-VSV-N (1:2000; Kerafast, Boston, USA). Finally, HRP-coupled goat-anti-mouse (1:5000; Jackson Laboratory #111-035-062) was added to the membranes for 45 min at room temperature. Blots were incubated with ECL solution, and chemiluminescence was recorded with an ImageQuant™ LAS4000 (GE Healthcare, Illinois, USA).

### Single-step growth kinetics

BHK-21 cells were seeded in 24-well plates (Corning Incorporated, New York, USA) at a density of 1 × 10^5^ cells per well. On the following day, cells were incubated with VSV-GP-Env variants or VSV-GP as a control at an MOI of 5 for 1 h at 37 °C. After five times washing with PBS, cells were supplemented with 1 ml of fresh medium. Supernatants were collected after incubation for the specified times and frozen for subsequent TCID_50_ assays.

### FACS staining for Env folding on 293T cells

In 6-well plates (Costar/Corning Incorporated, New York, USA), 1 × 10^6^ HEK293T cells were seeded per well and incubated overnight at 37 °C. On the following day, cells were infected with VSV-GP-Env variants or VSV-GP as a control at an MOI of 0.1. After 24 h, cell suspensions were collected and stained in U-bottom 96-well plates (Sarstedt, Nümbrecht, Germany) with Env-specific antibodies or the LCMV GP-specific Wen4 as control. Prior to staining with Wen4, cells were fixed with 1.5% formaldehyde for 15 min and subsequently washed once with FACS buffer (PBS supplemented with 1% FCS and 0.05% sodium azide). Cell pellets were re-suspended in primary antibodies diluted in FACS buffer VRC01 (1 µg/ml), F105 (10 µg/ml), PG16 (10 µg/ml), 447-52D (10 µg/ml), 10E8 (10 µg/ml), 17b(10 µg/ml), PGT151 (10 µg/ml), VRC07 (10 µg/ml), PGV04 (10 µg/ml), NIH46-46 (10 µg/ml), 39 F (1 µg/ml), b12 (10 µg/ml), PGT121 (10 µg/ml), PGT145 (10 µg/ml), 2G12 (10 µg/ml), or; Wen4 (1:10, hybridoma supernatant produced in-house) and incubated for 1 h at 4 °C. Cells were subsequently stained with secondary antibodies diluted in FACS buffer (donkey anti-human-Cy5, 1:200 diluted, Dianova, Germany or goat-anti-mouse-APC, 1:100 diluted, Invitrogen, USA) for 30 min at 4 °C. Finally, cells were washed using FACS buffer and fixed using 1.5% formaldehyde. Stainings with germline-specific antibodies (VRC01 germline, VRC07 germline, PGV04 germline; 25 µg/ml) were performed using directly Alexa Fluor 647-conjugated antibodies (Alexa Fluor 647 Protein Labeling Kit, A20173, Thermofisher Scientific, USA). Samples were analyzed with a FACS Canto II (BD Biosciences, USA) and FACS Diva software (BD Biosciences, USA). Each staining was performed in three independent experiments using duplicate samples. Geometric mean fluorescence signals for GP and HIV Env-specific antibodies were determined. To evaluate Env-specific antibody binding on vector-infected cells, the Env-specific signal was first normalized to LCMV GP staining to account for infection efficiency between the different vectors. Env-signals for VSV-GP control vector infected cells were subsequently set to 1, resulting in a representation of Env signal as fold-increase from VSV-GP.

### Tetramer staining of murine CD8^+^ T-cells

Peripheral blood was collected from the tail veins of immunized mice seven days post-immunization. Twenty microliters of blood were stained with 50 µl/tube H-2K^b^ VSV NP tetramer (MBL Life Sciences, Japan), diluted 1:25 in FACS buffer, for 20 min at 37 °C. Cells were washed and subsequently stained with surface marker antibodies anti-CD3 (PE-Cy7, 1:200), anti-CD8 (Pacific Blue, 1:750), anti-CD44 (PE-Cy5, 1:250), anti-CD62L (APC-Cy7, 1:500) (all BD BioSciences, New Jersey, USA), and anti-CD43 (FITC, 1:200) (BioLegend, California, USA) for 30 min at 4 °C. Erythrocytes were lysed twice using 500 µl of BD PharmLyse™ buffer (1:10, BD BioSciences, New Jersey, USA). Finally, samples were washed twice and fixed in 300 µl FACS fixing buffer (1.5% formaldehyde in PBS). Samples were analyzed with a FACS Canto II (BD BioSciences, New Jersey, USA) and 10,000 events in the CD3^+^CD8^+^-gate were recorded.

### Direct anti-HIV-1 Env enzyme-linked immunosorbent assay (ELISA)

High protein-binding ELISA plates (Costar/Corning Incorporated, New York, USA) were coated overnight at 4 °C with 1 µg/ml of the respective Env protein in PBS. On the next day, plates were blocked for 2 h at room temperature with 5% (w/v) fat-free milk powder in PBST. Heat-inactivated serum samples were serially diluted 3-fold in PBS plus 1% (w/v) BSA, starting with a 1:100 dilution and incubated at room temperature for 1 h. HRP-coupled goat-anti-mouse IgG or goat-anti-rabbit IgG antibodies (1:5000, Jackson Laboratory, Maine, USA) were added, and plates were incubated for 45 min at room temperature. To each well, 50 µl of KPL SureBlue™ TMB Microwell Peroxidase substrate was added, and the color reaction was stopped after 15–20 min with KPL TMB BlueSTOP™ solution (SeraCare Life Sciences, USA). Between all incubation steps, plates were washed five times with PBST. For subtype ELISAs, IgG-subtype specific antibodies were used: goat-anti-mouse-IgG1,-IgG2a and -IgG3 (1:5000, Jackson Laboratory, Maine, USA). Optical density (OD) was measured at 650 nm. A serum pool from non-immunized mice was measured on each plate and used to determine the background. Wells with an OD higher than 2× background and higher than background + 3 × SD were counted as positive. Endpoint titers were defined as the highest dilution factor that still yielded a positive result.

### Anti HIV-1 Env capture ELISA

Ni-NTA HisSorb plates (Qiagen) were coated with 350 ng of trimeric His-tagged gp140 antigen in 100 µL PBS per well and incubated overnight at 4 °C. After coating, the plates were washed three times with 200 µL PBST. Excess buffer was removed wells were blocked with PBS containing 5% BSA for 2 h at room temperature. After blocking, plates were washed three times with 200 µL PBST. Heat-inactivated serum samples were serially diluted 3-fold in PBS containing 1% BSA, starting with a 1:100 dilution and incubated for 2 h at room temperature. Plates were washed, and a HRP-coupled goat anti-rabbit IgG conjugate to HRP (1:10,000 in PBS containing 1% BSA) was added. After 2 h incubation at room temperature, the plates were further processed and endpoint titers calculated as described for direct anti-HIV-1 Env ELISA above.

### Anti-GP-1-ELISA

GP-1-IgG cells were cultivated for 6 days in TC-150 cell culture dishes to produce soluble GP-1-IgG^43^. Supernatants were harvested, and protein expression was verified with Western Blotting. High-protein-binding ELISA plates were coated overnight with goat-anti-human IgG antibody (Sigma-Aldrich, Missouri, USA), diluted 1:10,000 in 0.1 M NaHCO_3_. On the following day, plates were blocked with PBSTM for 3 h at room temperature. Afterwards, diluted GP1-IgG-containing supernatant was added to the plates for 1 h. Serum samples were diluted 3-fold starting with a 1:100 dilution in PBS plus 1% BSA (w/v) and incubated for 1 h at room temperature on the plates. The subsequent steps were carried out as described for the Anti-Env Enzyme-linked Immunosorbent Assay.

### VSV-GP-neutralization assay

Vero cells were seeded into F-bottom 96-well plates at a density of 1 × 10^4^ per well in 100 µl and were incubated overnight. On the following day, five-fold serial dilutions of heat-inactivated rabbit sera starting at a 1:10 dilution were prepared in duplicates, mixed with replication-deficient VSV-∆G-GP-SEAP particles and incubated at 37 °C for 1 h to allow neutralization of virus particles. Serum/virus mixes were then added to Vero cells, which were cultivated for an additional 24 h. As controls, four wells with virus only (no serum) and four noninfected wells were included. Finally, 40 µl of supernatants were transferred to a new 96-well plate and mixed with 200 µl of dissolved SIGMA Fast™ pNPP (Sigma Aldrich, Missouri, USA). Plates were incubated in the dark for 1 h at room temperature. OD at 405 nm was measured using an Epoch plate reader and Gen5 software (BioTek, Vermont, USA). OD-values were normalized to the virus only control and plotted with GraphPad Prism 9 (GraphPad Software Inc., California, USA). The nonlinear curve-fit function was utilized for the determination of ID_50_ values.

### HIV-neutralization assays

Neutralizing antibodies in rabbit serum were determined using lentiviral pseudotypes encoding a luciferase reporter as described previously^20^. PVs were produced by co-transfecting Env plasmids and the pSG3ΔEnv backbone (obtained from the AIDS Research and Reference Reagent Program) as previously described^52^. PVs displaying parental sC22 Env and parental sC23 Env are referred to as PV-sC22 and PV-sC23. PVs displaying glycosylation-modified sC22 KIKO and sC23 KIKO-derived Envs with N295 and N448 knocked in to enable binding of bnAb 2G12 and N-linked glycans at positions N276 and N460/N463 flanking the CD4 binding site, deleted by substitution of asparagine with glutamine (KO) to improve accessibility of the CD4 binding site, were referred to as PV-sC22 KIKO and PV-sC23 KIKO, respectively. Titers against PV-MW965.26, PV-SF162, PV-sC22, PV-sC22 KIKO, PV-sC23, and PV-sC23 KIKO Env pseudotypes were determined. Fifty percent inhibitory dilution (ID_50_ values) were determined using the nonlinear curve fit function in GraphPad Prism 9. Background was defined as 3 times the ID_50_ values obtained with pre-immune rabbit sera.

### Mouse immunization studies

Five to 6-week-old female C57BL/6JRj or BALB/cRj mice were purchased from Janvier Labs (Le Genest-Saint-Isle, France) and were kept in individually ventilated cages (IVC, environmental temperature 20–15 °C, nesting material and shelter provided for temperature regulation) in the animal facilities of the Medical University Innsbruck. Animals (*n* = 5 per treatment group) were immunized intramuscularly in weeks 0, 4, 8 and 12 with 1 × 10^7^ TCID_50_ of VSV-GP-Env or 20 µg Env protein plus 50 µg MPLA liposomes (Polymun Scientific, Austria). For intramuscular immunization, mice were briefly anesthetized using Forane inhalation (2–3% Forane with 2–2.5 L/min O_2_). The body weight was regularly monitored after all immunizations. Blood was collected from the tail vein and collected in non-coated microvettes for serum collection or EDTA-coated microvettes for FACS staining (Sarstedt, Nümbrecht, Germany). To obtain serum, blood samples were centrifuged at 8000 rpm 20 min. Mice were euthanized via cervical dislocation following Forane inhalation (2–3% Forane with 2–2.5 L/min O_2_).

### Rabbit immunization study

Female 5–6-week-old New Zealand White rabbits (strain code 052, Crl: KBL) were purchased from Charles River Laboratories (France) and were kept in the animal facilities of the Medical University Innsbruck. Animals (*n* = 4 per treatment group) received 4 intramuscular injections in weeks 0, 4, 12, and 20 containing 2 × 10^8^ TCID_50_ of VSV-GP-Env viruses or 40 µg Env protein plus 20 µg MPLA liposomes (Polymun Scientific, Austria). The body weight was regularly monitored. Blood was collected from the ear artery. To obtain serum, tubes were centrifuged twice at 2000 rpm for 10 min after allowing coagulation for 1 h. For euthanasia, animals were anesthetized by intramuscular injection of Ketamin/Xylazin (35 mg/kg and 5–10 mg/kg, respectively), blood withdrawal by heart puncture and intravenous overdosing of pentobarbital.

### Statistics

Statistical analyses were performed using GraphPad Prism version 9 (GraphPad Software Inc., California, USA) as indicated in the Figure legends. For comparison of more than two groups, nonparametric, non-paired one-way ANOVA with multiple comparison by Kruskal–Wallis was used. For comparison of two groups non-paired, nonparametric t-test (Mann–Whitney) was used.

### Ethics statement

Animal experiments were performed according to the requirements of the national animal experimentation law (“Tierversuchsgesetz”) and Austrian national authorities (Bundesministerium für Wissenschaft und Forschung, #BMBWF-66.011/0036-V/3b/2018 and #BMWFW-66.011/0148-WF/V/3b/2016) granted animal trial permissions.

## Supplementary information

Supplementary Materials

Supplementary Data 1

Supplementary Data 2

## Data Availability

All data supporting the findings of this study are available within the article and its Supplementary Information files. Further information or materials are available from the corresponding author upon reasonable request.
