# Supplementary material for: Heterologous combinations of VSV-GP and native-like trimers elicit autologous Tier 2 HIV antibodies in rabbits
Source: NPJ Vaccines. 2025 Dec 15;11:14. doi: 10.1038/s41541-025-01334-3 (PMC12816742; doi:10.1038/s41541-025-01334-3)
Supplement: Supplementary file 1 — Supplementary Materials [file 41541_2025_1334_MOESM1_ESM.pdf]

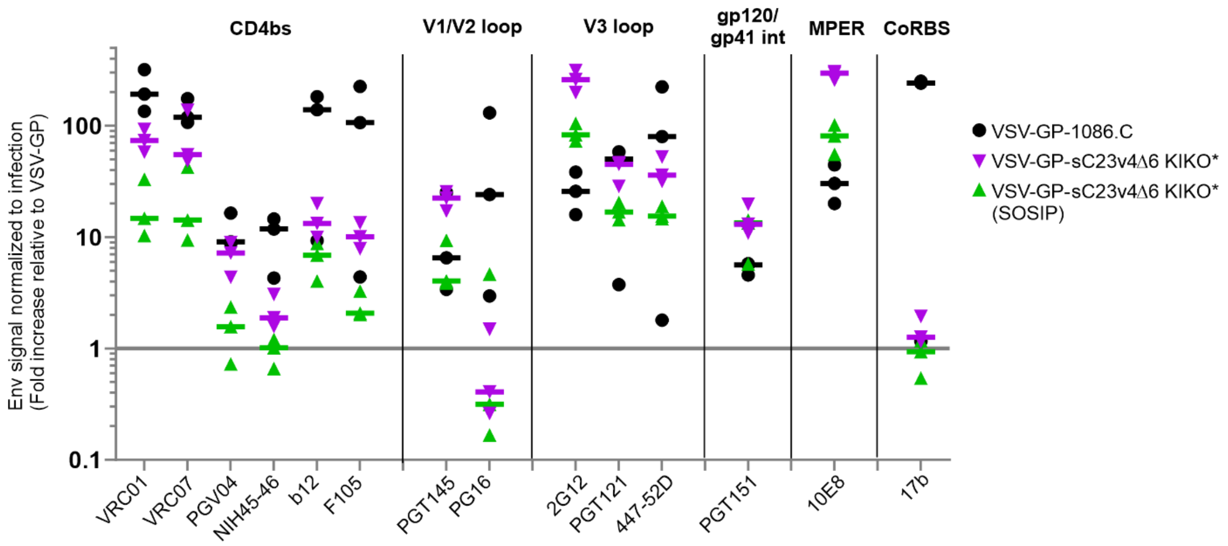

Supplementary Figure 1. NFL and SOSIP configurations of sC23v4Δ6 KIKO* fold similarly on infected cells. 293T cells were infected with VSV-GP-sC23v4Δ6 KIKO* variants in NFL (violet triangles) or SOSIP (green triangles) configuration or as controls a VSV-GP expressing the open trimer 1086.C (black circles) or an "empty" VSV-GP at an MOI of 0.1. On the next day, cells were stained with HIV Env-specific antibodies or the LCMV GP-specific Wen4 antibody as infection control and analysed via flow cytometry. Binding of Env-specific antibodies was normalized to infection (Wen4) and subsequently expressed as fold-increase relative to VSV-GP. Epitope regions on Env include the CD4 binding site (CD4bs), the V1/V2 loop, the V3 loop, the gp120/gp41 interface, the membrane-proximal external region (MPER) and the CD4-induced co-receptor binding site (CoRBS). Shown are results of three independent experiments as individual symbols and the medians as bold lines.

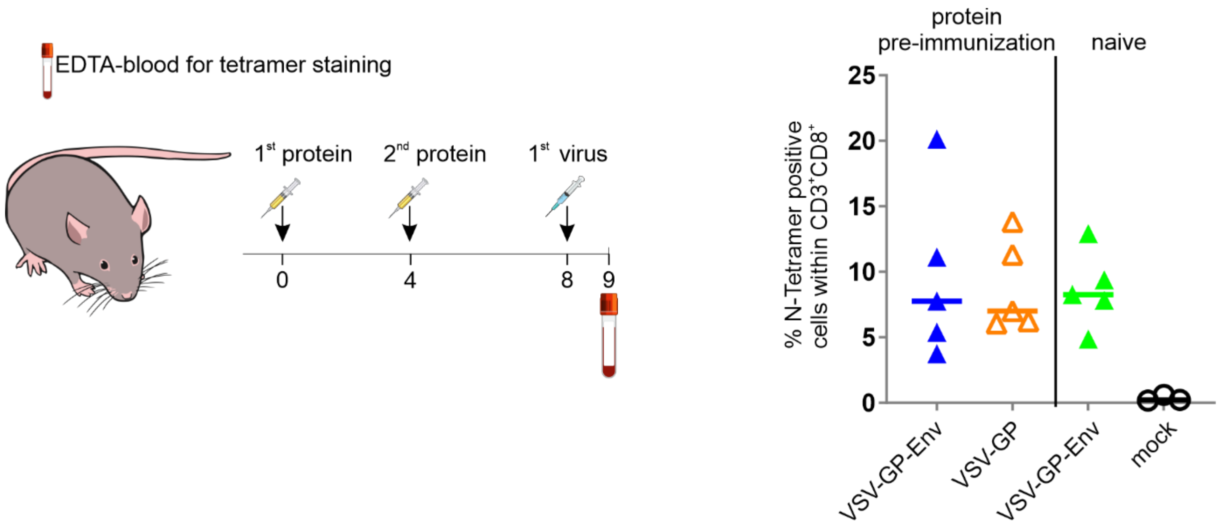

Supplementary Figure 2. Env-binding antibodies do not limit subsequent immunization with VSV-GP vectors presenting Env on the surface. (A) Female C57BL/6JRj mice were immunized twice with Env protein or left untreated. Animals were subsequently immunized with VSV-GP or VSV-GP-Env (vector variant with Env incorporated into the membrane of the vector particle) and vector-specific T cells were assessed via flow cytometry 7 days post infection. (B) Percentage of vector-specific T cells within $CD3^{+}CD8^{+}$ T cells in the blood were determined via tetramer staining using a tetramer against the immunodominant epitope in the vector backbone. Shown are median and individual animals for n = 5 per treatment group (n = 3 for mock).

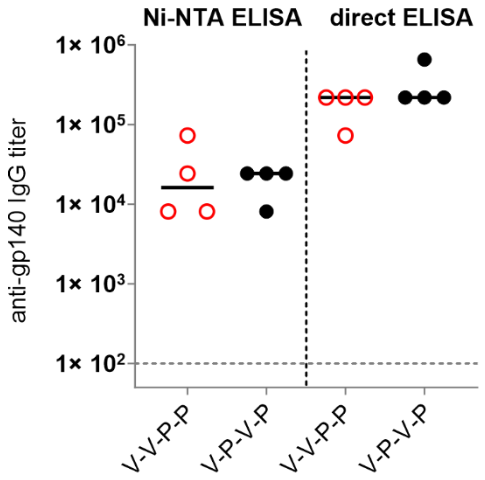

Supplementary Figure 3. Comparison of Ni-NTA capture ELISA and direct ELISA for detection of anti-gp140 antibodies. Rabbits were immunized either with two vector followed by two protein immunizations (V-V-P-P, classical heterologous immunization schedule) or alternating vector and protein immunizations (V-P-V-P, alternating heterologous immunization schedule). Sera from the final bleed (n=4 per group) were analysed for titers of gp140 binding antibodies by Ni-NTA capture ELISA (left) or direct ELISA (right). Shown are individual animals and median. Horizontal dotted line indicated limit of detection for the assay (1:100 serum dilution).

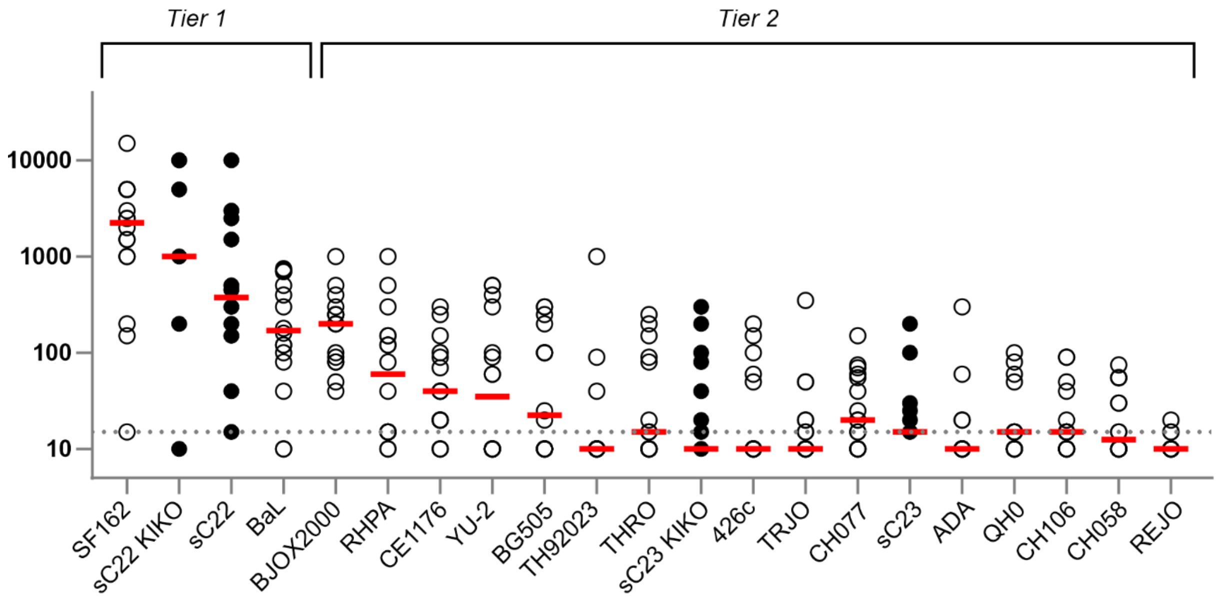

Supplementary Figure 4. Tier classification of sC22, sC22 KIKO, sC23 and sC23 KIKO pseudoviruses. Neutralization sensitivity of lentiviral pseudotypes was evaluated using a reference panel of sera from HIV-1-infected patients (n = 16). Envs from a reference virus panel with known Tier classification (open circles, NIH repository) as well as sC22, sC22 KIKO, sC23 or sC23 KIKO (closed circles) were analysed. Shown are ID_{50} (50 % inhibitory dilution) neutralization titers for individual sera and median (red bar). The detection limit was defined as 15 (dotted line), values below 15 were set to 10.

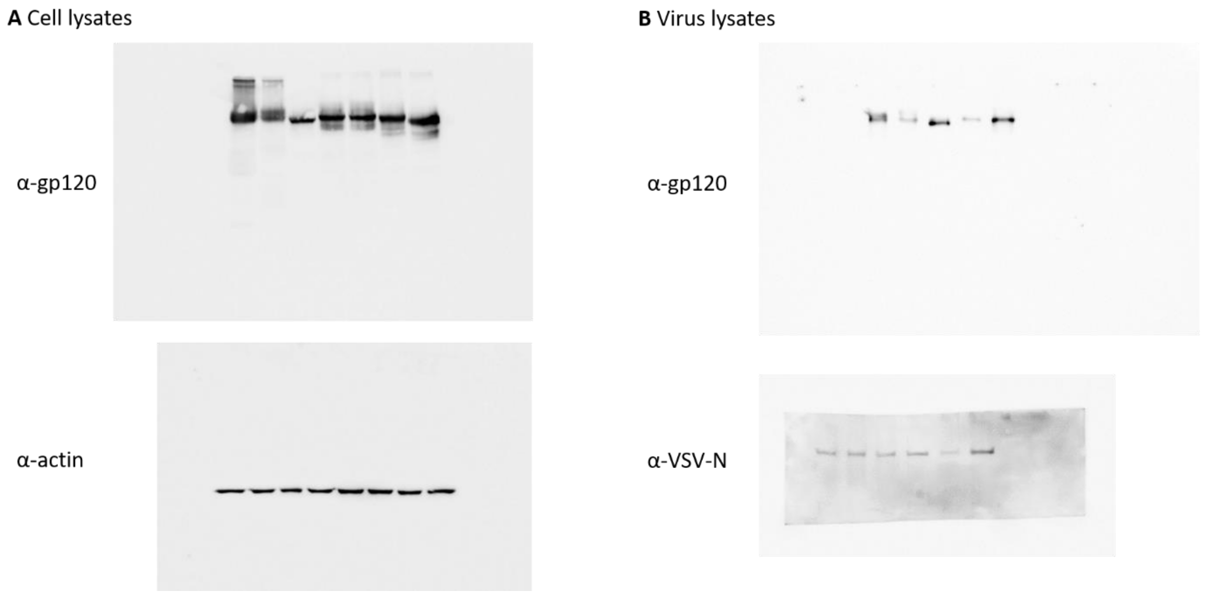

Supplementary Figure 5. Original western blots from Figure 1A. Uncropped images of western blotting membranes. (A) Cell lysates, (B) Virus lysates.

Supplementary Table 1. Recombinant VSV-GP constructs used in the study[#]

Vector	Class of Env	Env insert[§]	Furin cleavage site	Δ6 deletion
VSV-GP	no Env insert	-	-	-
VSV-GP-ZM96	open trimer	ZM96 gp140:G	mutated	no
VSV-GP-1086.C	open trimer	1086.C gp140:G	NFL	no
VSV-GP-sC22v4Δ6 KIKO*	native-like trimer	sC22v4 KIKO* gp140:GΔ6	NFL	yes
VSV-GP-sC23v4 KIKO*	native-like trimer	sC23v4 KIKO* gp140:G	NFL	no
VSV-GP-sC23v4Δ6 KIKO*	native-like trimer	sC23v4 KIKO* gp140:GΔ6	NFL	yes
VSV-GP-sC23v4Δ6 KIKO* (SOSIP)	native-like trimer	sC23v4 KIKO* gp140:GΔ6	SOSIP	yes

[#] Details on configuration of Env inserts are given in the Methods section; [§] gp140:G = HIV Env extracellular part (gp140) fused to the transmembrane domain and C-terminus of VSV-G; gp140:GΔ6 is an optimized version of the gp140 and VSV-G C-terminus fusion protein with enhanced accessibility of the 10E8 epitope (for details on design of antigens see Methods section).

Supplementary Data 1. Raw data for Figure 1C. 293T cells were infected with VSV-GP-Env variants at an MOI of 0.1 and 24 hours post infection, cells were stained with Env-specific antibodies or the LCMV GP-specific Wen4 as infection control. Secondary Cy5-coupled anti-human- or APC-coupled-anti-mouse IgG antibodies were used for detection. Binding of human neutralizing antibodies to VSV-GP-Env-infected 293T cells was quantitated by flow cytometry. Infection was done in three independent replicates and for each replicate duplicate samples were stained and analysed. Geometric mean fluorescence signals for Env-specific antibodies (VRC01, VRC01GL, F105, PGT151, 44-52D, b12, 39F, PGT145, VRC07, VRC07GL, NIH45-46, PGV04, PGV04GL, 10E8, 17b, 2G12, PGT121, PG16) and GP-specific Wen4 antibody are shown. Env-specific signals were normalized to infection (mean signal of Wen 4) for each virus. Data is shown in Figure 1C as fold-increase relative to VSV-GP.

Supplementary Data 2. Raw data for Supplementary Figure 1A. 293T cells were infected with VSV-GP-Env variants at an MOI of 0.1 and 24 hours post infection, cells were stained with Env-specific antibodies or the LCMV GP-specific Wen4 as infection control. Secondary Cy5-coupled anti-human- or APC-coupled-anti-mouse IgG antibodies were used for detection. Binding of human neutralizing antibodies to VSV-GP-Env-infected 293T cells was quantitated by flow cytometry. Infection was done in three independent replicates and for each replicate duplicate samples were stained and analysed. Geometric mean fluorescence signals for Env-specific antibodies (VRC01, F105, PGT151, 44-52D, b12, PGT145, VRC07, NIH45-46, PGV04, 10E8, 17b, 2G12, PGT121, PG16) and GP-specific Wen4 antibody are shown. Env-specific signals were normalized to infection (mean signal of Wen 4) for each virus. Data is shown in Supplementary Figure 1A as fold-increase relative to VSV-GP.
